# Mechanical properties of combined packable and high-filled flowable composite used for the fixed retainer: an in vitro study

**DOI:** 10.1186/s12903-024-04437-w

**Published:** 2024-06-10

**Authors:** Yasaman Alamdarloo, Seyed Ali Mosaddad, Farzaneh Golfeshan

**Affiliations:** 1 Orthodontic Research Center, School of Dentistry, Shiraz University of Medical Sciences, Shiraz, Iran; 2 Department of Research Analytics, Saveetha Institute of Medical and Technical Sciences, Saveetha Dental College and Hospitals, Saveetha University, Chennai, India; 3 Department of Conservative Dentistry and Bucofacial Prosthesis, Faculty of Odontology, Complutense University of Madrid, Madrid, Spain

**Keywords:** Adhesive, Biomedical and dental material, Fixed retainer, Composite

## Abstract

**Background:**

Clinicians often utilize both flowable and packable composites concurrently in bonding fixed retainers. Thus, this study aimed to assess the synergistic effect of these composites in the bonding process.

**Methods:**

This in vitro study divided specimens into three groups: flowable composite (nano-hybrid, Tetric N-Flow, Ivoclar Vivadent), packable composite (nano-hybrid, Tetric N-ceram, Ivoclar Vivadent), and combined use of flowable and packable composite. Shear bond strength (SBS), adhesive remnant index (ARI), and wire pull-out resistance were compared among the groups. Statistical analyses were conducted using ANOVA and Tukey tests to compare study groups. Additionally, Chi-square and Kruskal-Wallis tests were employed to analyze the ARI index among the groups.

**Results:**

ANOVA results indicated no statistically significant differences among test groups (*P* = 0.129) regarding SBS. However, a significant difference existed between flowable and packable composite groups (*P* = 0.01) regarding ARI scores. Among the study groups, flowable composite exhibited the highest frequencies of ARI scores of 1 and 2, whereas packable composite showed the highest frequency of ARI scores of 0. The combined group had higher frequencies of ARI scores of 0 and 1 compared to the flowable composite. The wire pull-out test revealed that the combined application of flowable and packable composite resulted in significantly lower detachments compared to the packable composite alone (*P* = 0.008). However, no significant differences were observed in the comparisons between the flowable-packable (*P* = 0.522) and combined-flowable (*P* = 0.128) groups.

**Conclusion:**

The combined use of flowable and packable composites for fixed retainers demonstrated adequate shear bond strength and ideal ARI scores, suggesting it as a suitable adhesive system for bonding orthodontic fixed retainers.

## Introduction

Orthodontic retention serves as the concluding stage of orthodontic treatment, focusing on maintaining teeth in their corrected positions following the completion of orthodontic tooth movement. However, teeth commonly tend to revert to their original positions due to the stretching of periodontal fibers, particularly those located at the tooth necks, including interdental and dentogingival fibers [1].

During the 1970s, fixed retainers were introduced as a preventive measure against the recurrence of lower incisor crowding following orthodontic treatments [2]. Lingually attached retainers are becoming increasingly popular among orthodontists. This preference is driven by their aesthetic appeal and the comfort they provide to patients, particularly during extended periods of wear [3, 4].

Fixed retainers offer several advantages, as supported by studies [5–7], including superior esthetic appeal, independence from patient cooperation, efficacy, and suitability for long-term retention. However, despite these benefits, certain drawbacks have been noted in the literature [8–10], such as their reliance on precise adhesive techniques, susceptibility to breakage, and potential to compromise oral cleanliness, leading to periodontal complications.

Multiple studies have explored the survival rates of bonded lingual retainers, with previously reported overall failure rates varying between 10.3% and 50% [8, 11, 12]. These failure rates differ across studies based on factors such as the materials used for retainer construction, the type of retainer, and the duration of follow-up. The failure of bonded fixed retainers can be attributed to several factors, including the detachment of the tooth-bonding interface, separation of the wire-bonding interface, breakage of the retainer wire, and unwanted torque movement of the teeth resulting from the retainer wire [11, 13–15].

Various adhesives have been specified for use in bonded retainers [16–18]. Conventional packable composites have conventionally been favored for fixed retainers due to their high filler content and resistance to abrasion. However, their application in the lingual region, where isolation is crucial, can be time-consuming [19]. In contemporary practice, the use of flowable composites with higher resin content, tailored for restorative dentistry, has been advocated for bonding fixed retainers. These flowable composites come in a range of formulations and viscosities to suit various applications. Notably, flowable composites incorporating nano-sized filler particles hold potential due to their higher filler content per unit volume and increased abrasion resistance compared to conventional micro-filled flowable composites, improving the material’s characteristics over conventional composites [20, 21]. Flowable composites eliminate the need for mixing during application. Their application syringes equipped with needle tips facilitate direct and precise injection of the composite, aided by its non-tacky nature. Additionally, no trimming or polishing is necessary, resulting in reduced processing time [22]. A considerable body of literature has explored various types of retainer wires, adhesive materials, and bonding techniques for fixed retainers [8, 13, 23–25]. In the bonding process of fixed retainers, securing the retainer wire adjacent to the lingual surfaces of the teeth prior to bonding poses a challenge for some practitioners. Methods such as finger pressure or pliers may raise concerns regarding stability. Various techniques have been introduced to address this issue, but many require additional laboratory or clinical steps, potentially prolonging the process. One method involves applying a small amount of flowable composite to hold the wire in place after the bonding agents have been applied to the lingual surfaces of the teeth. Subsequently, additional flowable or packable composites can be applied to both the teeth and fixed retainer wires to complete the bonding procedure [26].

Some major concerns regarding the bonding material include its ability to maintain adhesion over time without self-detaching. Thus, it necessitates a high bond strength. However, when intentionally debonded, it should leave minimal residues on the tooth’s surface to avoid more invasive and time-consuming cleanup procedures [27]. To date, a definitive adhesive for bonding fixed retainers has not been established. Previous studies [16, 25, 28] have primarily focused on evaluating and comparing the mechanical properties of fixed retainers bonded with either flowable or packable composites. To the best of the authors’ knowledge, no prior study has investigated the biomechanical properties of fixed retainer wires using both packable and flowable composites simultaneously.

In this in vitro study, the adhesive remnant index (ARI), shear bond strength (SBS), and wire pull-out were assessed for fixed retainers bonded with both flowable and packable composites and compared to those attached using each composite alone. The null hypothesis posited that the utilization of both flowable and packable composites together does not affect the studied outcomes.

## Methods

In this study, the samples were divided into three groups: flowable composite (Nano-Hybrid, Tetric N-Flow, Ivoclar Vivadent, Liechtenstein), packable composite (Nano-Hybrid, Tetric N-Ceram, Ivoclar Vivadent, Liechtenstein), and the combined use of flowable and packable composite. These groups were compared for SBS, ARI, and wire pull-out resistance. The study was approved by the Medical Ethics Committee of the Shiraz University of Medical Science (IR.SUMS.DENTAL.REC.1401.063) and is reported in accordance with ARRIVE guidelines.

### Specimens characteristics

Seventy-two extracted sound bovine incisor teeth were used for SBS and ARI evaluations. Based on a study by Reicheneder et al. [29], twelve pairs were allocated to each of the three study groups, resulting in a total of 72 samples. The minimum sample size was calculated to be nine pairs in each group using G*Power software with B = 0.2, α = 0.05, and a study power of 90%. However, to ensure higher accuracy, the minimum sample size was increased to 12 pairs in each group. Previous studies have validated the use of bovine teeth as a substrate for SBS testing [30]. These teeth were sourced from animals euthanized in a slaughterhouse for reasons unrelated to this study. After extraction, the teeth were rinsed with water and cleaned of any debris using a scaler. They were then stored in distilled water at 24 °C to maintain hydration [31]. Any teeth showing hypoplastic or anomalous enamel areas were excluded from the sample groups. The teeth were paired and embedded in chemically cured acrylic resin molds to simulate dental arch positioning and interdental contacts. The surfaces were oriented to allow for parallel cutting of the retainer in relation to the crown.

To conduct the wire pull-out test, a total of 192 cylindrical acrylic blocks were fabricated, each measuring 25 mm in width and 10 mm in depth. Custom molds compatible with the testing machine were utilized for this purpose. The minimum sample size for each of the three groups was determined to be 64, resulting in a total of 192 blocks, based on parameters including effect size (0.5), study power (90%), significance level (α = 0.05), and a non-centrality parameter (B = 0.2). These calculations were performed using G*power software. Consequently, each of the three test groups was allocated 64 blocks.

### Shear bond strength testing

**Fig. 1:**
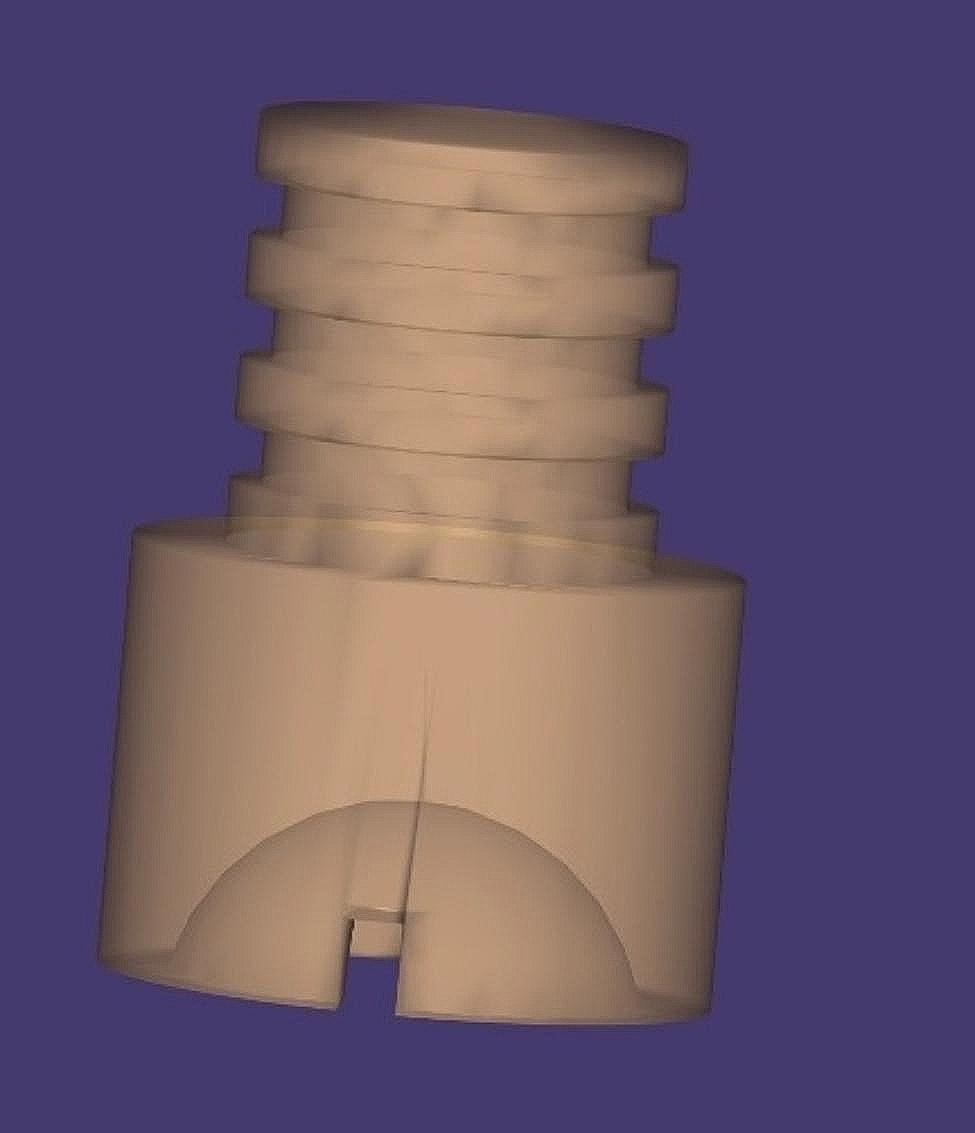
3D designed molds used for administration of composite resin

The samples were divided into three test groups, each comprising 24 teeth. A 37% phosphoric acid gel (3 M, USA, fluoride-free) was applied to etch the teeth’ lingual surface for 30 s, a standard etching time in orthodontic bonding [32]. After rinsing with water and air drying for 10 s, bonding resin (Tetric-N-bond, Ivoclar Vivadent, Liechtenstein) was applied and light-cured for 10 s in each test group. Subsequently, 15 mm lengths of passive retainer wire (American Orthodontics, three-strand, 17.5 twists) were bonded to the lingual surface of the teeth parallel to the acrylic base. Flowable composite was used for Group 1, packable composite for Group 2, and a combination of both composites for Group 3. The amount of composite used was standardized using a minidome-shaped mold (Fig. 1).

**Fig. 2:**
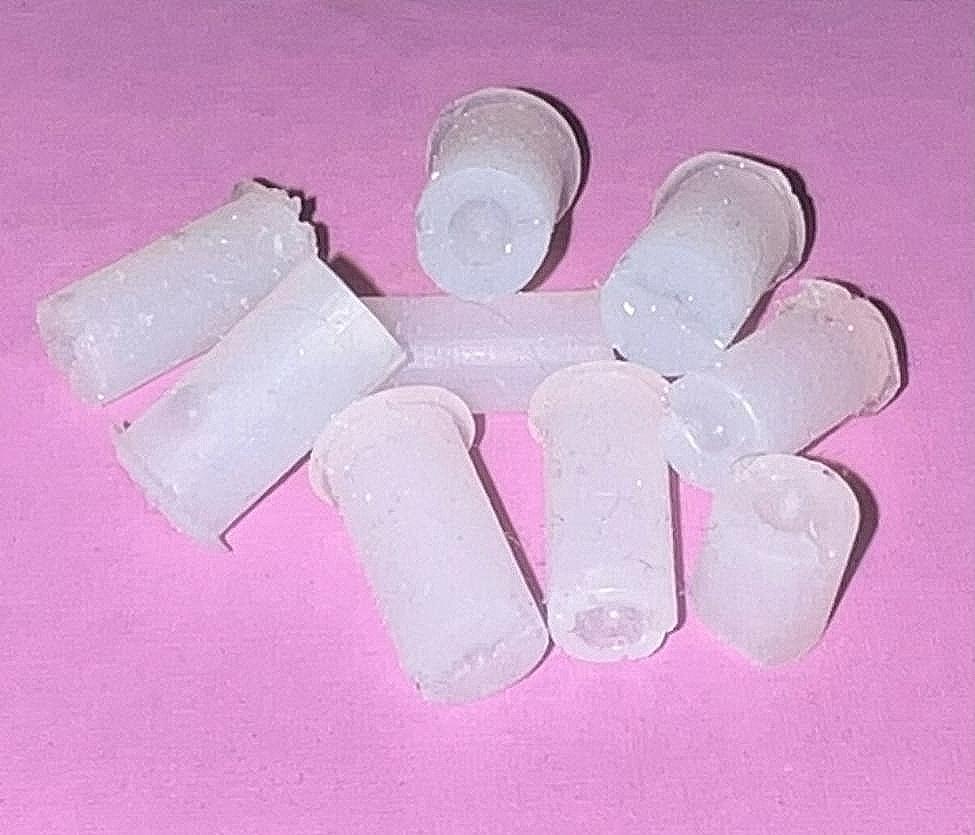
Mini molds used for the administration of composite resin

**Fig. 3:**
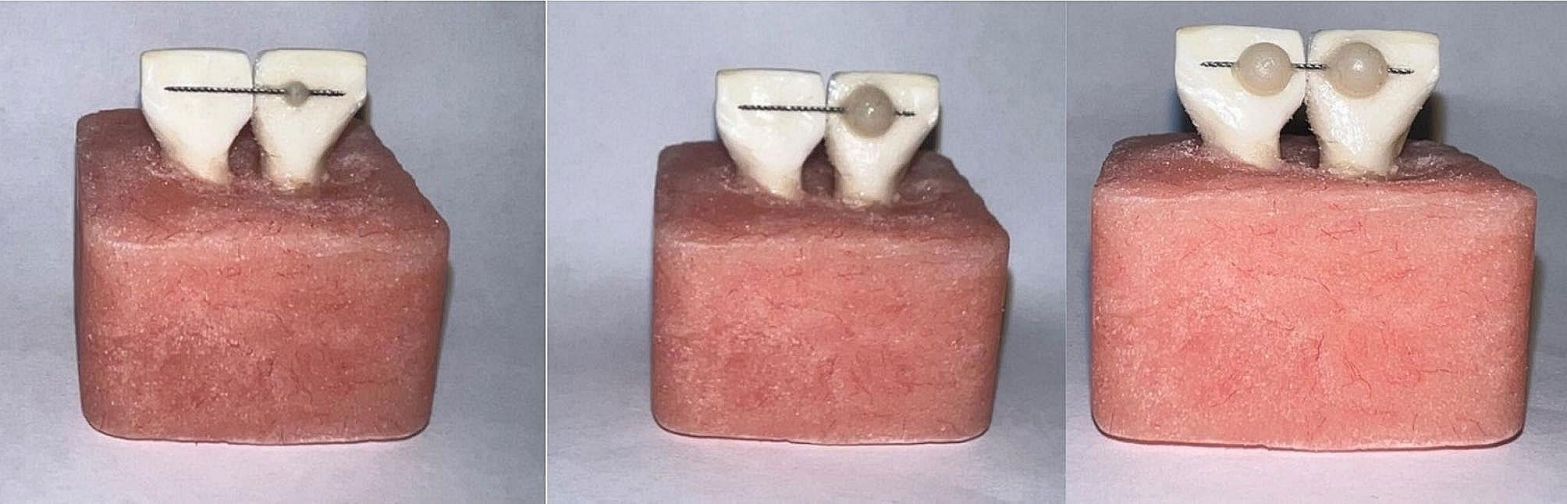
Preparing samples in the combined group

The composite resins were placed into a custom-made mini mold featuring an internal diameter of 4 mm and a height of 3 mm (Fig. 2). Within the mold, a groove facilitated the positioning of the composite to align with the wire at the center of the composite connection. The excess composite material was meticulously removed using a dental explorer, followed by curing with an LED curing light for 30 s—notably, the transparency of the mold allowed for effective light curing. In the case of utilizing both composites in Group 3, a flowable composite was initially packed and light-cured within a mini mold featuring an internal diameter of 2 mm and a height of 2.5 mm. Subsequently, the flowable composite was overlaid with a packable composite using a mini mold measuring 4 mm in diameter and 3 mm in height (Fig. 3). Post-application, the samples were de-molded and subjected to a second curing cycle lasting 20 s.

To maintain consistency in the bonding process, all retainers were standardized to a length of 12 mm. A flexible custom mold was employed to ensure adherence to standardized testing protocols, resulting in adhesive surfaces with a diameter of 4 mm and positioned 4 mm apart from each other. Each sample underwent a storage period of seven days in distilled water prior to testing.

**Fig. 4:**
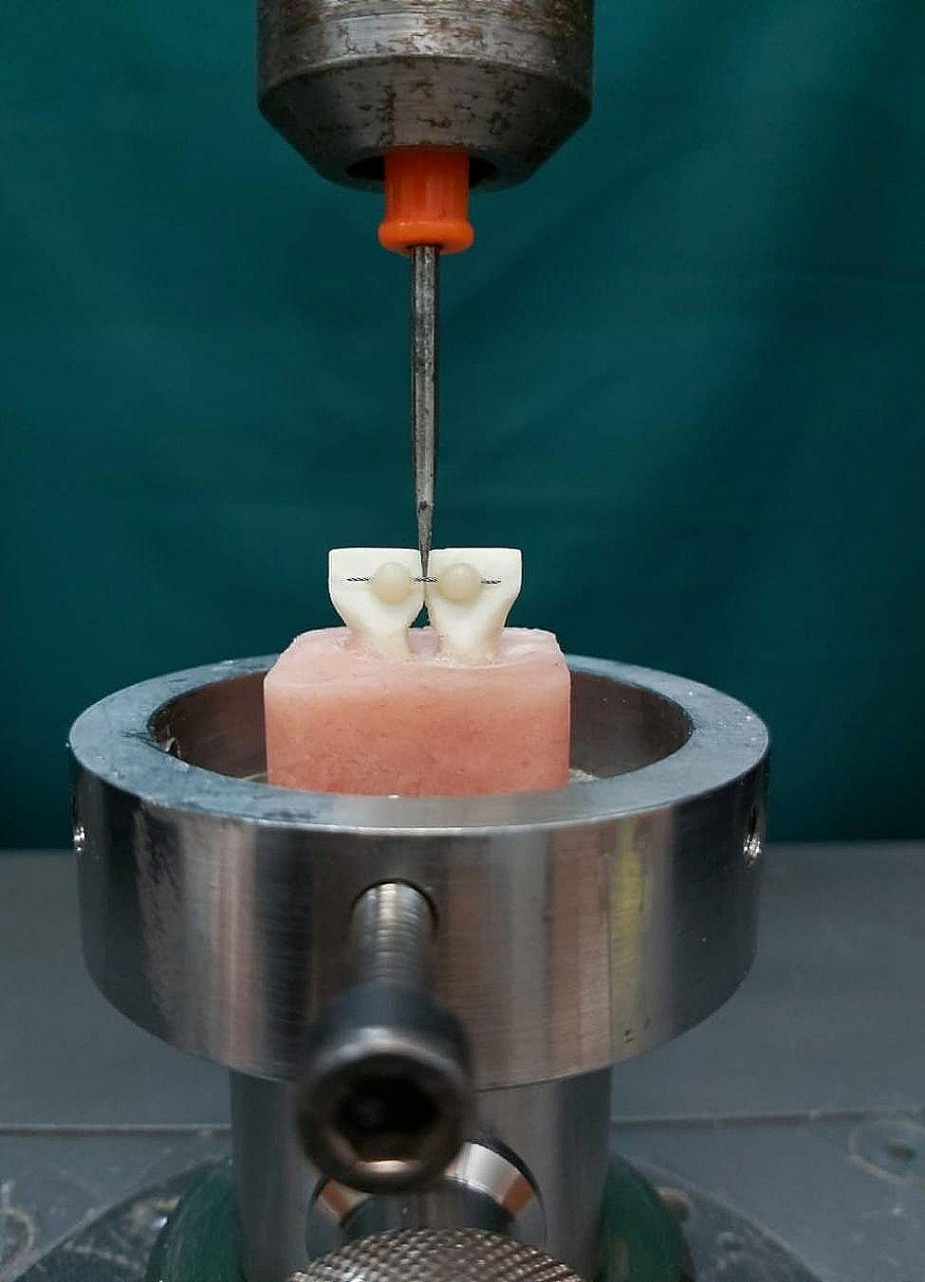
Measurement of shear bond strength on debonding using universal testing machine

The detachment procedure was conducted utilizing a Zwick Roell Universal Testing Machine-Z020, operating at a crosshead speed of 1 mm/min (Fig. 4). To simulate preliminary bite stress, the applied strain was directed along the occlusal-apical axis of the incisors. Consistent with established methodologies [28–30, 33, 34], the edge of the shear bar was positioned at the midpoint of the interdental segment, owing to its heightened sensitivity. Stress was incrementally applied to the wire until separation ensued, with the resulting SBS recorded in Newtons (N).

### Adhesive remnant index (ARI)

Following debonding, all teeth and retainers were examined using an optical microscope (Bestscope 300, BestScope Technology Co., Ltd., China) to assess the residual adhesive on the enamel surfaces. The quantification of adhesive remnants adhering to the teeth surfaces was carried out according to established guidelines for assessing the ARI [35]. The criteria were as follows:

- Score 0 = No adhesive left on the tooth.
- Score 1 = Less than half of the adhesive left on the tooth.
- Score 2 = More than half of the adhesive left on the tooth.
- Score 3 = All adhesive left on the tooth, with a distinct impression of the retainer.

### Wire pull-out

A small rectangle was crafted on the top of each block using a wire measuring 2.5 mm wide, 5 mm long, and 4 mm deep, representing the quantity of wire typically inserted in a bonded dental retainer. Additionally, a small groove measuring 1 mm in width was carved into the top surface of the block, extending across its entire diameter, with the intention of securing the wire in place. This groove was made approximately 2 mm deep in each test group to demonstrate the depth of penetration of both the wire and the composite material into the tooth surface.

**Fig. 5:**
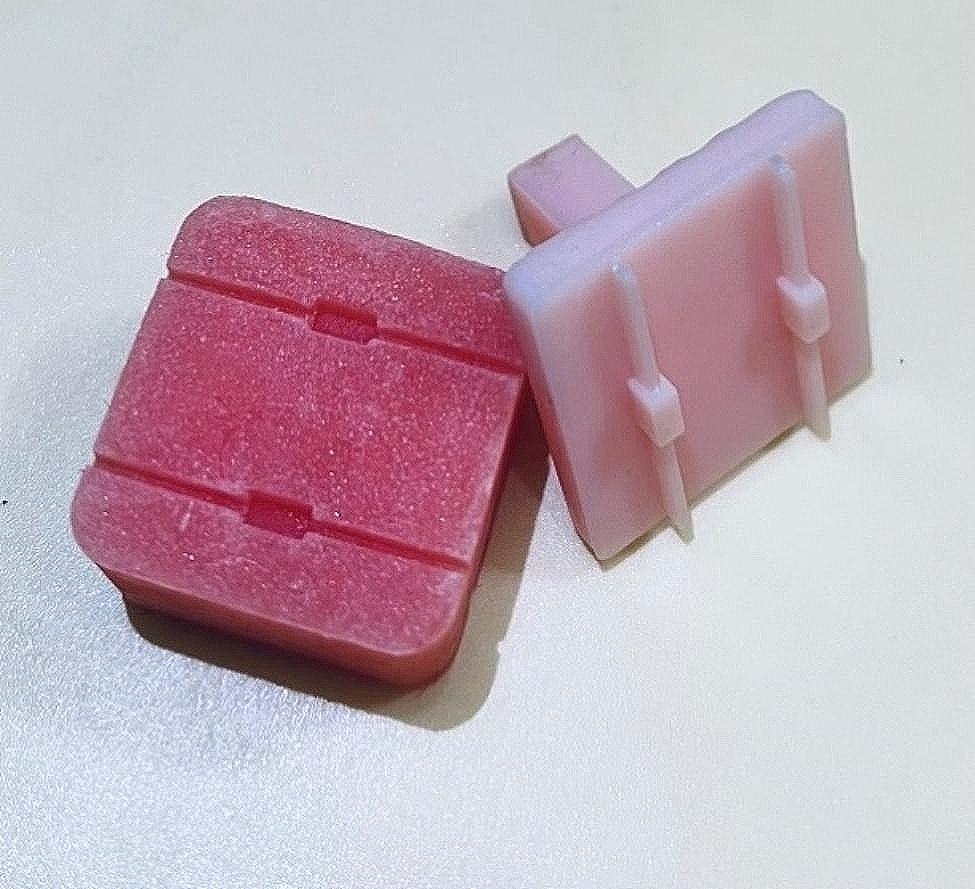
3D-designed stamp for wire pull-out test

**Fig. 6:**
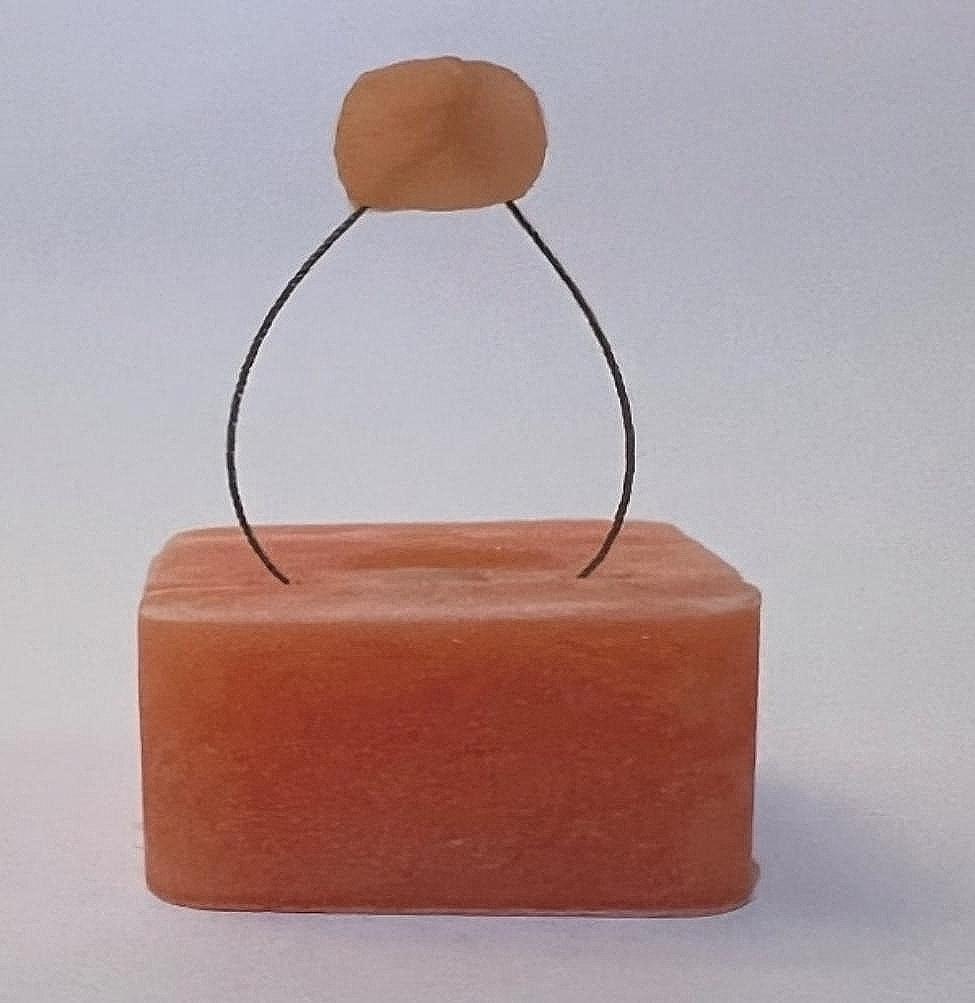
The sample used for the wire pull-out test

The fabrication of the rectangle and groove was accomplished using a stamp designed in 3D and printed with resin (Fig. 5). Subsequently, the upper surface of the block, formed by the stamp, was polished and prepared for bonding. Initially, any debris within the slot and center hole was manually removed. Then, the slot and hole were dried using compressed air. An 8.5-cm long three-strand rope was passively positioned at the bottom of the groove, followed by the passive placement of an 8.5-cm length three-strand wire at the base of the groove. A 3.5-cm length was marked on both sides of the wire, and a node was tied by two Matthew knots (Fig. 6). Subsequently, acrylic was poured onto the node to secure it in place during the test.

Following this, the wire was inserted into the specific composite material designated for testing. In Group 1, the wire was embedded in the packable composite, while in Group 2, it was embedded in the flowable composite. Group 3 involved a combination of both composite types. The void in the middle of the slot was filled with the test material. Special attention was paid to ensuring intimate contact between the plastic and the wall of the center hole of the slot without any obstruction from air bubbles. Any excess material was removed by the sculptor. Finally, the composite was treated with light for 30 s. To prepare Group 3, a flexible silicone mold with dimensions of 2.5 mm in width, 1 mm in length, and 2 mm in depth was used as a barrier against the spread of flowable composite throughout the cavity.

**Fig. 7:**
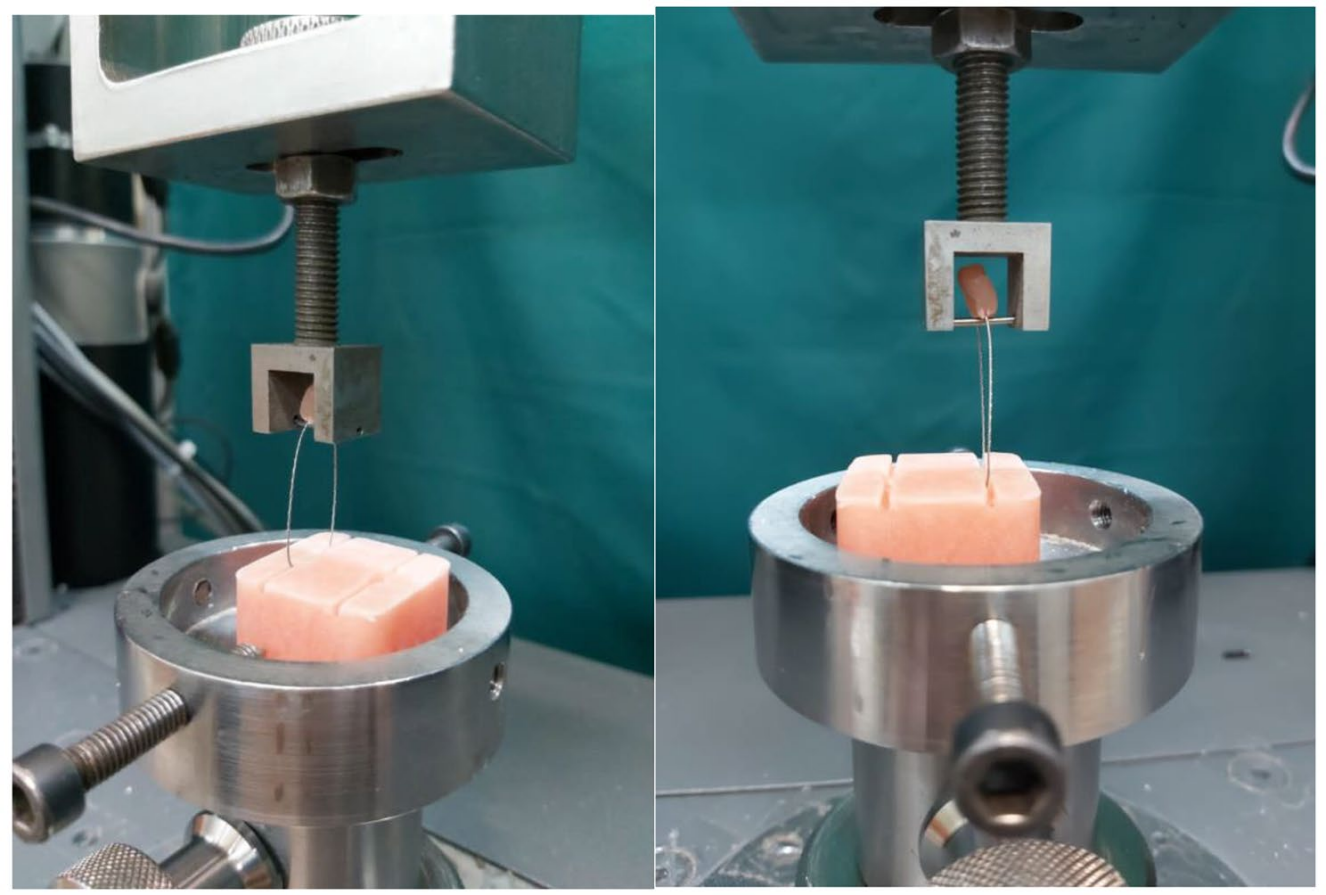
Wire pull-out test

The molds were positioned on both sides of the wire, after which the wire was embedded in flowable composite up to half of the mold’s depth, equivalent to 1 mm. Following the curing of the flowable composite, the molds were removed, and the remaining space, encompassing the former mold placement and extending 1 mm above the flowable composite, was filled with packable composite (Fig. 7). Subsequently, the ends of the wires were pulled and connected to enable fixation using the tension sensor fixing lever of the universal tester (Zwick Z020). This setup allowed for the application of force perpendicular to the dip cord’s length to initiate the movement of the rope. Testing for damage was conducted by moving the crosshead at a speed of 10 mm per minute [36]. The force required to extract the wire from the device was measured in Newtons (Fig. 7).

### Statistical analysis

The average and variation values for each study group were calculated using the collected data from the experimental groups. ANOVA was employed to assess significant differences among the groups, followed by the application of the Tukey HSD range test to confirm any observed disparities with a 95% confidence level. Furthermore, the Chi-square test was used to investigate variations in ARI scores across different groups, and pairwise comparisons were performed using the Kruskal-Wallis test. Data analysis was performed using SPSS software (IBM Corp. Released 2013. IBM SPSS Statistics for Windows, Version 22.0. Armonk, NY: IBM Corp.)

## Results

Table 1 presents the mean, standard deviation, and results of the ANOVA test for shear bond strength across all groups. The ANOVA analysis indicated no statistically significant differences between the test groups (*P* = 0.129).

**Table 1:** Means and standard deviations of sbs testing, and (anova results) statistical results

Groups	*N*	Min	Max	Mean	Standard Deviation	*P* value
Flowable	12	82.68	211.43	148.42	50.24	0.129
Packable	12	39.71	186.12	104.69	49.66	
Combined	12	45.28	241.83	145.76	70.23	

Table 2 displays the frequency distribution of ARI scores across different groups. The results of the nonparametric Chi-Square test demonstrated statistically significant differences among the test groups (*P* = 0.01). Subsequently, a pair-wise comparison (Kruskal-Wallis test) test was conducted. Among the study groups, flowable composite demonstrated the highest frequencies of ARI scores of 1 and 2, while packable composite exhibited the highest frequency of ARI scores of 0. The combined group showed higher frequencies of ARI scores of 0 and 1 compared to the flowable composite. Based on the results of pair-wise comparison, only the difference between flowable and packable composites was found to be significant (*P* = 0.01) (Table 3).

**Table 2:** Frequency distribution of the adhesive remnant index (ARI) of the three groups evaluated

Groups	ARI Scores*					*P* value
	0	1	2	3	Total	0.01
Flowable	2	4	5	1	12	
Packable	11	1	0	0	12	
Combined	5	5	0	2	12	

*0 = No adhesive left on the tooth, 1 = Less than half of the adhesive left on the tooth, 2 = More than half of the adhesive left on the tooth, 3 = All adhesive left on the tooth, with a distinct impression of the bracket mesh

**Table 3:** Pairwise comparison of groups for ARI scores

Groups	*P* value
Packable-combined	0.74
Packable-flowable	0.01
Combined-flowable	0.46

Table 4 presents the wire pull-out values for different groups. The ANOVA results indicated significant differences between the test groups. Subsequently, Tukey’s HSD test was employed for pairwise comparisons. The results demonstrated that the combined application of flowable and packable composite yielded significantly lower debonding compared to the packable composite alone (*P* = 0.008). However, no significant differences were observed in the comparisons between the flowable-packable (*P* = 0.522) and combined-flowable (*P* = 0.128) groups,, as illustrated in Table 5.

**Table 4:** Means and standard deviations of wire pull-out test and statistical results

Groups	*N*	Min	Max	Mean	Standard Deviation	*P* value
Flowable	64	94.10	275.00	156.83	49.69	0.01
Packable	64	100.00	294.00	169.30	50.46	
Combined	64	82.60	227.00	134.27	36.75	

**Table 5:** Post hoc tests for pairwise wire pull-out comparison

Primary Group	Comparison Group	*P* value
Flowable	Packable Combined	0.522 0.128
Packable	Flowable Combined	0.522 0.008
Combined	Flowable Packable	0.128 0.008

## Discussion

After orthodontic therapy, teeth often tend to relapse to their original positions due to insufficient time for periodontal tissues to reshape. It is crucial to maintain the achieved tooth positioning throughout the reorganization process. To achieve this, the utilization of removable or fixed retention systems is necessary [1]. The necessity for reliable maintenance of tooth alignment following orthodontic intervention is undisputed from a clinical perspective. The bonded wire retainer is a multifaceted apparatus subject to numerous forces emanating from varying directions [34]. Orthodontists now favor new bonding materials for retainers, which are bonded either between canine teeth or between premolar teeth to maintain the alignment of lower anterior teeth [8]. This method, which has shown effectiveness and aesthetic appeal, is gaining recommendation among specialists who advocate for using flowable composites to attach lingual retainers [37]. Many clinicians now utilize a combination of both flowable and packable composites during the bonding of fixed retainers. Flowable composites offer ease of application with their needle tips, allowing clinicians to apply them to the tooth effortlessly. On the other hand, packable composites boast high filler content, resulting in enhanced abrasion resistance and reduced shrinkage. Consequently, the present study was designed to include a third group employing both flowable and packable composite materials in the bonding procedure to evaluate the synergistic effects of these two composites.

In the SBS analysis of the study, the mean values ranged from 39.71 to 82.68 N, with Group 1 (Tetric-N-flow, Ivoclar Vivadent, Liechtenstein) exhibiting the highest shear bond strength. However, the difference in mean strength among the groups was found to be statistically insignificant. This finding is consistent with a study by Radlanski and Zain [28], who observed a maximum shear bond strength of 64.3 N for Tetric-N-Flow bonded assemblies, which was higher than for Tetric-N-Flow bonded Heliosit® Orthodontic packable composite (Ivoclar Vivadent GmbH, Ellwangen, Germany) assemblies. Singh et al. also noted that the wire-composite combination of Respond (Ormco Corp., Orange, CA, USA) dead-soft wire with Tetric-N-Flow composite exhibited the maximum shear bond strength, surpassing that of G-aenial™ Universal Flo, microfilled hybrid composite (GC America Inc.) [33]. The enhanced shear bond strength of the flowable composite can be attributed to its filler content. The filler content of packable composite is 80% by weight, whereas that of Tetric-N-Flow is 63.8% by weight. With decreasing filler content, viscosity decreases, suggesting that Tetric-N-Flow would have superior flowability and wettability compared to the packable composite, thereby contributing to increased bond strength [33]. Contrary to our findings, Reicheneder et al. [29] and Al-Nimri et al. [23] reported that Transbond™ LR (3 M™ Unitek, Solventum, Germany) showed higher SBS compared to Tetric-EvoFlow™ (Ivoclar Vivadent, Liechtenstein), Stick®ORTHO flow (Stick Tech Ltd., Turku, Finland), and Filtek Z250 (3 M™ Unitek, Solventum, Germany). In these studies, the wires used were Bond-A-Braid™ (Reliance Orthodontic Products Inc., Itasca, USA) and GAC Wildcat® Twistflex Wire (Ortho-Care Ltd., Bradford, UK), respectively, which may account for the differences in the results observed. This suggests that additional factors, such as the diameter of the wire or the number of turns it possesses, could serve as determinants of shear bond strength [29].

Reynolds [38] discovered that materials utilized in orthodontic treatment should possess sufficient strength to endure forces ranging between 6 and 8 N. Similarly, Waters et al. noted that the typical force exerted during biting ranges from 3 to 18 Newtons [39]. In the current research, all tested retainer systems exhibited bond strengths exceeding expectations, indicating their suitability for practical use.

Accurately assessing the ARI is crucial as it plays a significant role in selecting an orthodontic adhesive. The ARI is among the most frequently employed methods for evaluating the bond quality between composite and tooth surfaces, as well as between composite and orthodontic appliances [40]. Studies have suggested that variations in ARI values signify differences in bond strength between the enamel and the adhesive for various adhesive systems. Nevertheless, there is a trend towards adhesive systems that leave minimal adhesive residue on the tooth surface, facilitating easier and safer removal of residual resin [19]. In a study conducted by Cooke et al., differences in ARI scores were investigated. It was proposed that the flexible wires (measuring 0.016 × 0.022 inches and three-stranded 0.0175 inches) placed between teeth were subjected to tension, causing them to bend. This bending likely resulted in cracks forming in the material adjacent to the wire, particularly at the junction between the wire and the material, ultimately leading to bond failure between the wire and the material [34].

In the current study, a notable difference was observed between the flowable and packable composite groups in terms of ARI. Flowable composite exhibited a higher frequency of ARI scores of 1 and 2, while packable composite showed a higher frequency of ARI scores of 0. In the combined group, there was a greater frequency of ARI scores of 0 and 1. There has long been debate among clinicians regarding whether bond strength between the tooth surface and the composite or easier debonding with less risk of enamel damage is more important [41]. A score of 2 or 3 on the ARI index indicates bond failure at the adhesive and retainer wire interface, leaving more composite on the tooth surface, which can lead to a more time-consuming debonding process and a higher risk of enamel damage [27, 42]. Conversely, scores of 0 or 1 on the ARI index signify bond failure at the enamel and adhesive interface, possibly due to incorrect bonding procedures or inadequate bond strength from the adhesive system [43]. In such cases, the debonding process is typically easier and less destructive to enamel integrity [42]. However, if clinicians must choose between easier debonding and optimal strength, the latter is generally preferred [42].

Considering that a good orthodontic biomaterial should ideally have an ARI value of 1 or 2 and a bond strength in the range of 5–50 MPa to withstand chewing forces [44], the combined group’s values, with a mean of 45.28 for shear bond strength and a high frequency of an ARI score of 1, fall within these optimal ranges, suggesting an adequate system.

Based on the wire pull-out test results, the packable composite exhibited statistically significantly higher detachment values than the combined group. However, no statistically significant differences were observed between flowable and packable composites. In contrast, it has been revealed that non-flowable composite for retainers generated stronger forces than flowable composite when cured with a light-curing device [22]. Packable composites require a stronger force for removal from surfaces due to their higher filler content and superior physical properties. Conversely, flowable resins typically exhibit lower strength compared to regular composites, but they offer greater flexibility due to their lower filler content. In materials composed of different components, this reduced filler content decreases the strength required to extract the wire [22].

Within the scope of our investigation, both shear bond strength and wire pull-out were quantified through laboratory measures. However, it is important to acknowledge that the present study may be limited in its ability to fully replicate clinical conditions [44]. An additional limitation worth noting is the use of bovine teeth as substitutes for real human samples. Empirical investigations may be necessary to assess the influence of salivary enzymes, the physiological mobility of dental structures, biomechanical stressors induced by tongue movement and chewing, as well as the effects of dental biofilm and mineralized deposits. These factors are crucial components of in vivo conditions that warrant further exploration.

## Conclusion

Within the limitations of this study, all tested groups exhibited adequate bonding strength suitable for clinical application. The combined application of flowable and packable composites for bonding fixed retainers demonstrated satisfactory shear bond strength and adequate ARI scores, with minimal detachment observed at the composite-wire interface (cohesive fracture). These findings suggest that the combined use of flowable and packable composites serves as a viable adhesive system for bonding orthodontic fixed retainers.

## Data Availability

The data supporting this study’s findings are available from the corresponding author upon reasonable request.

## Abbreviations

SBS: Shear bond strength
ARI: Adhesive remnant index
N: Newtons
